# Multiplex Genome Engineering Methods for Yeast Cell Factory Development

**DOI:** 10.3389/fbioe.2020.589468

**Published:** 2020-10-29

**Authors:** Koray Malcı, Laura E. Walls, Leonardo Rios-Solis

**Affiliations:** ^1^Institute for Bioengineering, School of Engineering, The University of Edinburgh, Edinburgh, United Kingdom; ^2^Centre for Synthetic and Systems Biology (SynthSys), The University of Edinburgh, Edinburgh, United Kingdom

**Keywords:** CRISPR/Cas technology, multiplex genome engineering, simultaneous genome integration, delta integration, rDNA clusters, yeast cell factory development, *Saccharomyces cerevisiae*, non-conventional yeasts

## Abstract

As biotechnological applications of synthetic biology tools including multiplex genome engineering are expanding rapidly, the construction of strategically designed yeast cell factories becomes increasingly possible. This is largely due to recent advancements in genome editing methods like CRISPR/Cas tech and high-throughput omics tools. The model organism, baker’s yeast (*Saccharomyces cerevisiae*) is an important synthetic biology chassis for high-value metabolite production. Multiplex genome engineering approaches can expedite the construction and fine tuning of effective heterologous pathways in yeast cell factories. Numerous multiplex genome editing techniques have emerged to capitalize on this recently. This review focuses on recent advancements in such tools, such as delta integration and rDNA cluster integration coupled with CRISPR-Cas tools to greatly enhance multi-integration efficiency. Examples of pre-placed gate systems which are an innovative alternative approach for multi-copy gene integration were also reviewed. In addition to multiple integration studies, multiplexing of alternative genome editing methods are also discussed. Finally, multiplex genome editing studies involving non-conventional yeasts and the importance of automation for efficient cell factory design and construction are considered. Coupling the CRISPR/Cas system with traditional yeast multiplex genome integration or donor DNA delivery methods expedites strain development through increased efficiency and accuracy. Novel approaches such as pre-placing synthetic sequences in the genome along with improved bioinformatics tools and automation technologies have the potential to further streamline the strain development process. In addition, the techniques discussed to engineer *S. cerevisiae*, can be adapted for use in other industrially important yeast species for cell factory development.

## Graphical Abstract

Overview of key approaches used for multiplex genome engineering for yeast. **(A)** Delta sequences and rDNA cluster found in the yeast genome can be targeted for multi-copy integration. **(B)** For donor DNA delivery, multiple linearized plasmids or multiple oligonucleotides can be used to target multi-loci. **(C)** Multiple sgRNAs can be used to target specific regions in the yeast genome. CRISPR/Cas system can also be used coupling with other approaches demonstrated here to increase integration efficiency. **(D)** Synthetic sequences can be placed into the genome in advance so that they serve as entry points, “gates,” to the genome. Pre-placed gates can be used for both higher integration efficiency and fine-tuning by controlling of copy numbers of the genes of interest.

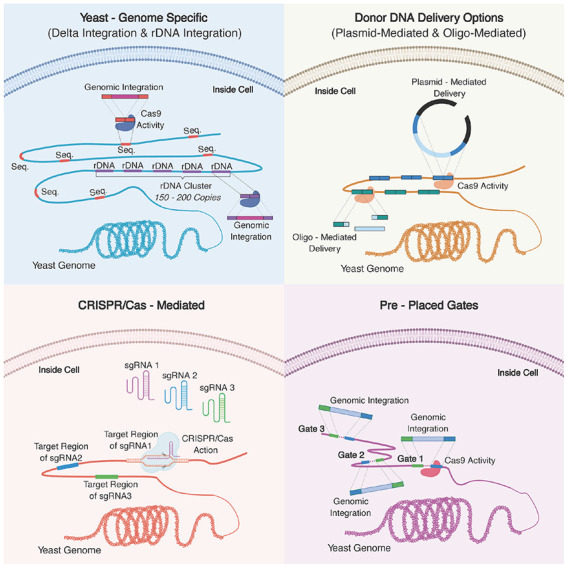

## Introduction

*Saccharomyces cerevisiae* (Baker’s yeast) has been exploited by humans for millennia for the production of fermented foods and beverages. However, the species has gained substantial interest as a model platform for the renewable production of valuable chemicals in recent times (Nowrouzi et al., 2020; Walls et al., 2020). Such chemicals have extensive applications across the agricultural,

energy, food and drinks, cosmetics and pharmaceutical industries among others (Fazilah et al., 2018; Wong et al., 2018). As chemical and natural biosynthetic routes are limited, there is a great demand for stable microbial cell factories for their production (Li and Borodina, 2014; Gustavsson and Lee, 2016; Wong et al., 2016) *S. cerevisiae* is a particularly attractive candidate for biotechnological applications as it can be grown to high densities on inexpensive media in relatively low-cost industrial scale fermenters. Its genetic and physiological features are well characterized (Hong and Nielsen, 2012; Buchholz and Collins, 2013; Li and Borodina, 2014) and relevant tools and knock-out libraries are widely available for straightforward strain manipulation (Jensen and Keasling, 2014).

As affordability of commercial DNA synthesis and high-throughput tools for cloning and DNA assembly has radically improved in recent times, genetic manipulation at genome-scale has become increasingly possible (Widłak, 2013; Diggans and Leproust, 2019). With this, understanding of gene function along with the identification of novel functional genetic parts and genome-level molecular organizations has increased dramatically (Rodenburg, 2018; O’Donnell et al., 2020). Consequently, simultaneous multi-enzyme expression and multi-loci genome integration can now be achieved, accelerating metabolic engineering and strain development efforts.

Numerous multiplex genome manipulation and gene integration techniques have been developed for *S. cerevisiae*. For example, a TALEN-based method, TALEN-assisted multiplex editing (TAME), was developed to improve ethanol tolerance in the species. This is a multiplex genome editing tool which makes use of the GC and TATA boxes in the *S. cerevisiae* genome (Zhang et al., 2015). The method was subsequently extended to improve stress tolerances in *S. cerevisiae* strains (Gan et al., 2018). However, TAME relies on protein-DNA interactions (Nemudryi et al., 2014) and the design of new proteins for each application, which has hindered its widespread use as a multiplex genome editing tool. As a result of its increased flexibility and effectiveness, CRISPR/Cas technology has been employed in most recent multiplex genome integration studies. In addition, traditional genome integration methods such as plasmid-based integration and recombineering are increasingly being coupled with CRISPR/Cas technology to enhance their efficiency. Yeast-specific multiplex genome integration methods including integration in ribosomal DNA (rDNA) clusters and delta integration have also been adapted for use with CRISPR/Cas9.

Multiplex genome engineering is widely considered critical to expediting the strain development process in *S. cerevisiae* and research into multiplex engineering technologies is accelerating at an unprecedented rate, as highlighted in a number of recent reviews. Stovicek et al. (2017), for example, addressed different approaches for the simultaneous expression of multiple gRNAs and the use of donor DNAs to enable multiplexing. Auxillos et al. (2019) also reviewed several multiple genome editing methods with a focus on optimizing bio-production in *S. cerevisiae*. They mainly highlighted CRISPR-independent multiplexing approaches with high-throughput screening techniques to select desired individuals in a yest cell library produced by multiple genome engineering.

This review considers recent studies in which traditional genome editing methods such as delta integration, rDNA clusters and plasmid-based integration were coupled with CRISPR/Cas technology for enhanced efficiency. An overview of recent advancements in multiplex genome engineering tools for *S. cerevisiae* focusing on multiple integrations is provided. Novel approaches such as pre-placed sequences which act as gates into the genome are also discussed. In addition, the role of state-of-the-art automation platforms in further expediting multiplex genome editing and strain development is reviewed. Significant recent progress in multiple gene deletion, disruption, up-regulation and down-regulation techniques for the optimization of metabolic pathways and biomanufacturing are also discussed. Finally, the extension of multiplex engineering techniques to engineer the genomes of non-conventional yeasts is considered.

## Optimization of CRISPR/Cas9 for Multiplex Genome Engineering

CRISPR/Cas, first introduced in 2012 (Jinek et al., 2012), is widely recognized as one of the most promising and revolutionary genome engineering tools. The technique offers a number of advantages over traditional methods, namely selective, marker-free integration, precise, targeted dsDNA cleavage and straightforward target fragment insertion via homologous recombination.

Soon after the discovery of the CRISPR/Cas system for genome engineering, a multiplex CRISPR (CRISPRm) method capable of inducing multiple double strand breaks (DSB) across the genome was developed (Ryan et al., 2014). Through the fusion of a self-cleaving ribozyme to the sgRNA sequences, duplex integration efficiency was improved 12-fold. The CRISPR/Cas9 system was expressed by a single vector containing the DNA sequences encoding the Cas9 protein sgRNA and HDV ribozyme sequences. However, even with the optimized method triplex integration efficiency was below 20% (Ryan et al., 2014).

**FIGURE 1:**
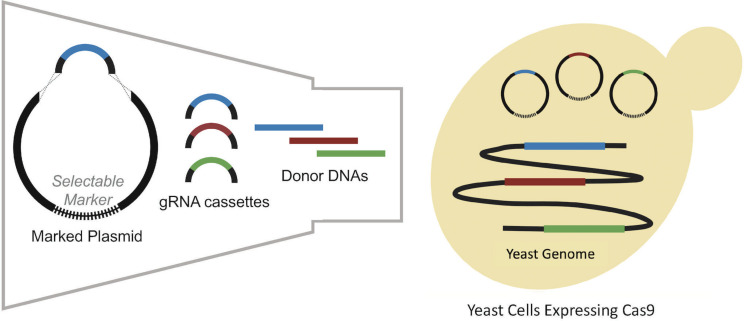
Modular delivery of the CRISPR-Cas9 system adapted from Horwitz et al. (2015). Different gRNA cassettes are assembled into plasmids by gap repair mechanism. After co-transformation of plasmids containing different gRNAs, donor DNAs are integrated into the genome by homologous recombination to repair DSBs formed by Cas9 which is expressed in cells.

As integration efficiency was found to be a key bottleneck, research focus shifted toward the development of alternative CRISPR/Cas9 delivery systems with enhanced multiplex integration efficiency. This included a modular gRNA sequence delivery method, in which linearized plasmid DNA expressing a selective marker and multiple linear gRNA sequences were co-transformed into a strain expressing Cas9 (Horwitz et al., 2015). Each gRNA sequence targeted a different locus and contained 500 bp of flanking homology to the ends of the plasmid DNA facilitating homologous recombination *in vivo* (Horwitz et al., 2015), as summarized in Figure 1. With this modular gRNA delivery method, successful triplex integration was achieved in 64% of transformants. However, subsequent attempts to simultaneously insert 11 genes distributed between six different fragments from the muconic acid pathway, resulted in a dramatic decrease in efficiency to 4.2%. The low efficiency of the method was attributed to its reliance on a large number of homologous recombination events between fragments and the host genome along with the large size of the target construct (24 kb).

## Yeast Genome-Specific Multiplex Integration

Although several multiplex genome engineering methods have been available for decades, low efficiency was a major bottleneck, especially as cassette size was increased. More recently, traditional genome integration techniques have been adapted for coupling with the flexible and effective CRISPR/Cas tools. In this section several traditional multiplex engineering strategies and work to enhance their efficiency using CRISPR/Cas are discussed.

### Delta Integration and Its Coupling With CRISPR/Cas9

**FIGURE 2:**
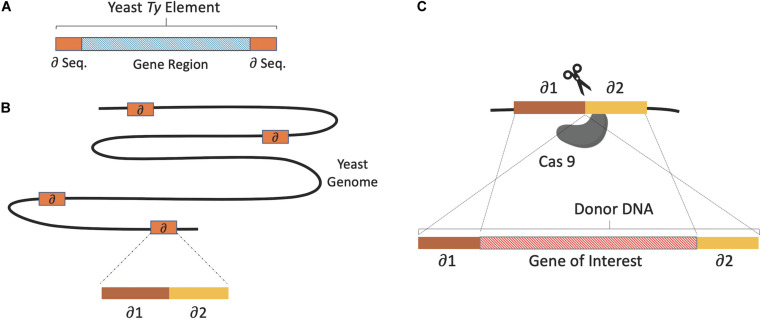
**(A)** The basic structure of *Ty* elements, gene region coding 2 proteins flanked by δ sequences. **(B)** Multi copies of δ sequences dispersed on different yeast chromosomes. **(C)** Donor DNA flanked by homologous δ sequence fragments used for integration via homologous recombination. Where CRISPR/Cas9 (scissors) is used, Cas9 cuts the δ sequence in the middle to form a DSB, which is subsequently repaired using donor DNA.

The simultaneous integration of multiple genes into the yeast genome was first attempted in the late 1980s. Multiple copy and multiple loci integration of human β-endorphin and mouse α-amylase coding genes into the *S. cerevisiae* genome was achieved via delta (δ) integration (Sakai et al., 1990). This involved the use of the yeast retrotransposon, Ty, a mobile genetic element with identical replication and integration systems as metazoan retroviruses (Krastanova et al., 2005). Ty has two terminal direct repeats or δ sequences and it is estimated that over 80 copies of the δ sequence are present in the yeast genome. The heterologous gene sequences were inserted into the δ sequence facilitating chromosomal integration at the δ sequence via homologous recombination, as illustrated in Figure 2. As the mobile elements of *S. cerevisiae* can exceed 150 copies, dispersed across different chromosomes, δ sequence targeting is an efficient method for multiple copy and multiple loci integration (Alastair Grace and Carr Patrick McHugh, 2018; Rowley et al., 2018).

Recently, the δ-integration method was coupled with CRISPR-Cas (Di–CRISPR) making use of gRNA sequences to target and induce DSB at the δ sequences (Shi et al., 2016). This facilitated simultaneous integration of multiple copies of large linearized DNA sequences. A 24 kb cassette encoding pathways for xylose utilization and (R, R)-2,3-butanediol (BDO) production each comprised of three heterologous genes along with green fluorescent protein (GFP) was efficiently integrated via Di–CRISPR. With the optimized method 18-copy integration of the 24 kb cassette was achieved in a single step. Although efficiency decreased as cassette size was increased from 8 to 24 kb, the copy number achieved for the 24 kb cassette was 5.9-fold higher using Di-CRISPR compared to traditional δ-integration (Shi et al., 2016).

Huang and Geng (2020) also employed a CRISPR-mediated δ-integration method to integrate a 2,3-butanediol (2,3-BDO) biosynthesis pathway into the *S. cerevisiae* genome. For this, three genes responsible for 2,3-BDO production, α-acetolactate synthase (*alsS*) and α-acetolactate decarboxylase (*alsD*) from *Bacillus subtilis*, and the native 2,3-butanediol dehydrogenase (*BDH1*) were used (Ramos et al., 2000; Schmidtke et al., 2012). The pathway genes along with an enhanced green fluorescent protein reporter, more than 4 kb size in total, were assembled *in vivo* via homologous recombination and integrated using both CRISPR-mediated and traditional δ-integration. Using CRISPR-mediated δ-integration a maximum of 25 and average of 13.4 copies of the 2,3-BDO pathway was achieved, compared to an average of 7.5 copies using conventional δ-integration. The resulting average 2,3-BDO titer was almost two-fold higher in CRISPR-mediated strains at 1.10 g/L compared to 0.56 g/L in the strains engineered through conventional δ-integration.

An alternative approach known as CRISPR/Transposon gene integration (CRITGI), which directly targets the Ty1 retrotransposon sites rather than the terminal δ sequences has also been recently developed (Hanasaki and Masumoto, 2019). In the study a CRISPR plasmid including Cas9 and a sgRNA targeting the Ty1 loci was co-transformed with a Ty1 containing plasmid (pTy1). Following Cas9 mediated DSB induction in the Ty1 sequences of the pTy1 plasmid and genome, integration of the pTy1 plasmid was achieved via homologous recombination. Using this approach 12 copies of the pTy1 plasmid DNA were successfully integrated into the yeast genome.

To summarize, targeting multi-copy transposable elements existing in the genome offers several benefits. Episomal expression via plasmids is ubiquitous for multi-copy expression of heterologous genes. However, segregational plasmid instability hinders stable expression of the heterologous genes (Da Silva and Srikrishnan, 2012). Stable multi-copy chromosomal integration at multiple loci across the genome is therefore an excellent alternative method for the overexpression of genes of interest. In addition, as transposable elements contribute toward genome evolution (Bleykasten-Grosshans and Neuvéglise, 2011), disruption can minimize the risk of genomic alterations. This facilitates the development of strains with enhanced genetic stability.

### rDNA Clusters and Its Coupling With CRISPR/Cas9

**FIGURE 3:**
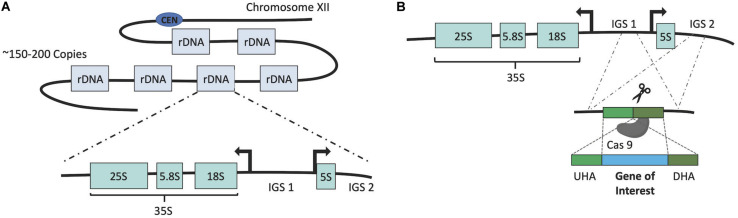
**(A)** Dispersion and structure of rDNA genes. **(B)** Intergenic regions (IGS1, IGS2) can be targeted to integrate GOI as they are dispersed among multi-copy rDNA genes. Where CRISPR/Cas9 (scissors) is used, Cas9 cuts a particular point in the IGS.

The development of the δ-integration was concurrent with that of an alternative method which made use of rDNA repeats (Lopes et al., 1989). As a eukaryote, the genome of *S. cerevisiae* contains tandem repeats of rDNA sequences, which are responsible for transcription of ribosomal RNAs. These rDNA repeats are the most abundant gene found on chromosome XII (150∼200 copies). Each repeating unit contains 35S and 5S rRNA genes along with the intergenic regions IGS1 and IGS2, which contain regulatory elements (Kobayashi and Sasaki, 2017; Cutler et al., 2018). As a result of such features, the rDNA locus was found to be an appropriate target for multi-copy integration of both homologous and heterologous genes within the *S. cerevisiae* genome. The method was first employed to integrate native phosphoglycerate kinase (PGK) and Mn^2+^-dependent superoxide dismutase (SOD) coding genes along with a heterologous thaumatin gene originating from *Thaumatococcus daniellii* (Lopes et al., 1989). The flexible CRISPR/Cas9 technology has also been coupled with multi-copy integration into rDNA cluster through the development of CRISPR–Cas9-assisted multiplex genome editing (CMGE) (Wang et al., 2018). Figure 3 describes the general features of integration into rDNA clusters and its coupling with CRISPR/Cas9.

This was first used to engineer the thermotolerant, methylotrophic yeast, *Ogataea polymorpha* (Wang et al., 2018). The method was successfully applied for multiplex gene knockouts, multi-copy (MC) and multi-locus (ML) integration of the yeast. Multi-copy integration (CMGE-MC) was subsequently adapted for editing *S. cerevisiae*. This involved the co-transformation of gRNA sequences targeting the intergenic regions (IGS1) of rDNA repeats with an episomal vector and donor DNA expressing the gene of interest (GOI) into a *S. cerevisiae* strain constitutively expressing Cas9. The resulting colonies harbored up to 10 GFP copies and were stable for 55 generations.

As rDNA genes are responsible for both ribosomal function and structure, Chiou and Armaleo (2018) investigated the effects of alterations on rDNA clusters using CRISPR/Cas9. The effect of such mutations on cellular fitness was investigated through the simultaneous introduction of point mutations in the Cas9 cutting site of all rDNA copies. A 57 bp intron from lichen fungus *Cladonia grayi* was also integrated. Unlike yeast, this species belongs to one of several phyla that possess introns within their rDNA gene sequences. Yeast strains harboring point mutations and rDNA introns survived and mutations were stably inherited as they were spliced out correctly from the rRNA transcripts during rRNA processing. It was further demonstrated that the approach could be applied to integrate uniformly distributed insertions or mutations on all rDNA copies within the genome.

In a recent study the δ sequence and rDNA site integration methods were combined to engineer an efficient cytosolic isobutanol biosynthetic pathway in *S. cerevisiae* (Park and Hahn, 2019). Firstly, *AlsS* was integrated using a plasmid-based δ-integration method. Endogenous ketol-acid reductoisomerase (*ILV5*) and dihydroxy-acid dehydratase (*ILV3*) coding genes with modified N-terminal sequences to facilitate cytosolic localization were subsequently integrated into the rDNA sites. The resulting engineered strain produced 263 mg/L of isobutanol, 3.3-fold higher than the control strain, which expressed the pathway via plasmids. A strain expressing a cytosolic isobutanol pathway with an enhanced production titer was successfully developed without CRISPR/Cas9. However, a maximum copy number of four was achieved for both δ and rDNA site integration. The coupling of CRISPR/Cas9 with rDNA, CMGE (Wang et al., 2018), δ-integration (Shi et al., 2016; Huang and Geng, 2020) or CRITGI (Hanasaki and Masumoto, 2019) resulted in substantial improvements in integration efficiency and copy number. The use of a combination of CRISPR-mediated approaches therefore has the potential to improve isobutanol titers further.

rDNA clusters are a good alternative to delta integration as there are hundreds of copies of rDNAs in the yeast genome. It has also been observed that the chromosomal integrations can be stably inherited over 50 generations (Wang et al., 2018). However, rDNA genes are essential for the cell as they are responsible for transcription of rRNAs. Even though there are intergenic regions between the genes in rDNA cluster (see Figure 3A), IGS1 contains an origin of DNA replication (rARS) while IGS2 includes both the replication fork barrier (RFB) and a bidirectional RNA polymerase II-dependent promoter (Kobayashi and Sasaki, 2017). In addition, it has been revealed that IGS1 is also responsible for maintenance of nucleolar stability along with stabilization of rDNA repeat number (Cahyani et al., 2015). The disruption of even a few rDNA repeats among the hundreds of copies gained through evolutionary processes can therefore affect cell fitness and hinder strain development.

### Efficient Regions in the Yeast Genome for Gene Integrations

Several studies have shown that placing heterologous genes near autonomously replicating sequences (ARSs) within the genome has a positive effect on their expression rate. ARSs can promote transcription-factor activity associated with DNA replication initiation (Flagfeldt et al., 2009; Wu et al., 2019) and therefore may influence gene expression. A recent study by Apel et al. (2017) focused on the development of a CRISPR/Cas9-based toolkit for the efficient introduction of genetic modifications in *S. cerevisiae*. The researchers characterized 23 loci, 23 gRNA targets, within the yeast genome focusing their integration and expression efficiencies for a model gene encoding a GFP reporter protein. Of these loci, 16 were selected from the regions near ARSs, and the others were near several genes. It was reported that ten of the ARS-close gRNAs showed 100 % or close to 100 % integration efficiency, further demonstrating the positive influence of ARSs. Wu et al. (2017) also integrated red fluorescent protein (RFP) into 1044 locations scattered over 16 chromosomes of the yeast genome to observe position effects on heterologous gene expression. The researchers revealed that some regions have positive effect on gene expression whilst others, particularly those close to telomere or centromeres, can decrease expression rate. The integration locus is therefore critical to optimal gene expression and efficient regions such as those identified by Apel et al. (2017) and Wu et al. (2017) are likely to be good initial targets for multi-loci integrations in the yeast genome. However, further research into this and the development of alternative target regions are needed.

## Donor DNA Delivery Options

### Plasmid-Based Multiple Integration

Although episomal plasmids offer a straightforward means of heterologous gene expression in *S. cerevisiae*, large fluctuations in plasmid copy number occur. Yeast integrating plasmids can overcome such instability challenges. However, despite their relative stability compared to episomal plasmids, there is still a high risk of chromosomal rearrangements if multiple tandem insertions are used, which can lead to loss of the introduced genes (Jensen et al., 2014). The plasmid based EasyClone method was developed to facilitate the simultaneous stable integration of multiple genes into the genome of *S. cerevisiae* (Jensen et al., 2014). A vector set was produced by combining the advantages of uracil-specific excision reaction-based cloning technique (USER cloning) and the Cre-LoxP marker recycling method. Three fluorescent protein genes and promoters were inserted into three different USER integration cassettes containing one or two genes. Uracil containing primers were used to form overlapping fragments after USER^TM^ enzyme treatment (Nour-Eldin et al., 2006). A different auxotrophic selection marker was used for each cassette, each flanked with LoxP sites. The selection markers could therefore be looped out by Cre recombinase mediated recombination without losing the integrated fluorescent protein genes, facilitating selection marker recycling. Of 16 clones resulting from the triplex integration, 44% successfully exhibited triple fluorescence. Although simultaneous integration into three loci was reported in this approach, selection markers and their recycling are important features of this method. Therefore, the need for higher integration efficiency without using selective pressure made alternative approaches necessary.

**FIGURE 4:**
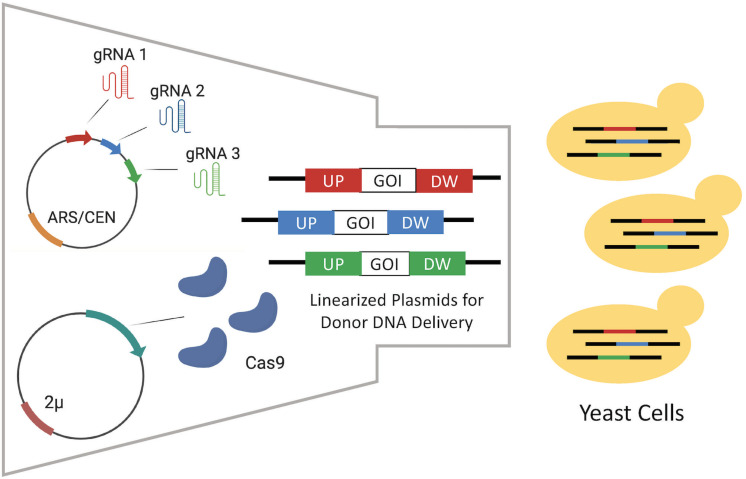
Episomal delivery of CRISPR system via the CrEdit method adapted from Ronda et al. (2015). Different gRNAs are expressed by gRNA expression plasmids for the multiplex integration of different donor DNAs on EasyClone integrative vectors. It was detected that the episomal expression of Cas9 performed better than the Cas9 expressed in the chromosome. Each different gRNA molecule guides Cas9s to form DSB in their target region. Next, the host repairs those DSBs via HR using linearized plasmids including upstream (UP) and downstream (DW) homologous arms.

As introduction of DSB by CRISPR/Cas9 significantly enhances integration efficiency through homology-directed repair (HDR) (Liu M. et al., 2019), a CRISPR/Cas9 mediated genome editing (CrEdit) method was developed. This involved coupling the highly efficient CRISPR/Cas9 system with the convenient EasyClone method (Ronda et al., 2015). Two methods were compared, in the first Cas9 and the multiple gRNA sequences targeting the different EasyClone integration sites were expressed from ARS/CEN based and 2 μ episomal plasmids, respectively (Figure 4). In the second, Cas9 was chromosomally integrated and gRNA expressed from a linearized integrative vector. Donor DNA sequences were delivered via integrative linearized EasyClone vectors. Three donor DNA sequences flanked with homology arms with lengths of either 60, 110, and 500 bp were compared. As expected, the DNA with the 500 bp homology arms was integrated with the highest efficiency. The integration efficiency was significantly higher for all three DNA sequences with the plasmid-based system at 99, 90, and 98% efficiency compared to 19, 3, and 9% for the chromosomally integrated Cas9 system, respectively. Using CrEdit, three genes (*BTS1*, *CrtYB*, and *CrtI*) from a β-carotene pathway were subsequently integrated into the yeast genome simultaneously. Of the resulting transformants, 84% successfully produced the characteristic orange pigment. A β-carotene pathway comprised of three genes with a total size of 17.5 kb was constructed efficiently in *S. cerevisiae*.

Similarly, EasyClone and CRISPR/Cas9 methods were coupled for the development of a *cis*,*cis*−muconic acid (CCM) pathway in *S. cerevisiae* (Kildegaard et al., 2019). The method was first tested by performing a triplex integration of three different fluorescent proteins (GFP, YFP, RFP), this was successfully achieved with an integration efficiency of over 70%, 1.6-fold higher than that obtained in the original study using EasyClone alone (Jensen et al., 2014). The subsequent construction of the CCM pathway involved the multiplex integration of genes from three different species, *Podospora anserina*, *Klebsiella pneumoniae*, and *Candida albicans*. Using CRISPR/Cas9 mediated EasyClone integration an engineered *S. cerevisiae* strain was constructed, cultivation of which yielded a CCM titer of 400 mg/L (Kildegaard et al., 2019). A combination of EasyClone vectors and CRISPR/Cas9 has also been used for multiplex gene knock-outs in an alternative yeast species, *Yarrowia lipolytica* (Holkenbrink et al., 2018). Comparison between CRISPR-aided vector integration and original EasyClone method in which CRISPR/Cas was not used clearly demonstrates that the induction of DSB can increase multi-gene integration efficiency.

The nature of the promoter initiating transcription has a profound impact on gene expression. The activation, inactivation or even replacement of native promoters with synthetic alternatives are therefore important factors to consider when fine-tuning the expression of a metabolic pathway (Baral et al., 2018; Hwang et al., 2018). Multiplex manipulation techniques targeting promoter optimization therefore have the potential to expedite the development of a microbial pathway with favorable gene expression levels. The plasmid-based multiple integration method, mpCRISTAR, was developed for simultaneous multiple promoter replacement (Kim et al., 2020). This technique combined transformation-associated recombination (TAR), a cloning-based genome manipulation method which makes use of the high homologous recombination rate of *S. cerevisiae*, with CRISPR/Cas9 (Kouprina and Larionov, 2016). Using mpCRISTAR, four CRISPR plasmids each expressing a gRNA and one of four auxotrophic selection markers (*URA3*, *HIS3*, *MET15*, and *TRP1*) were co-expressed in *S. cerevisiae*. This facilitated the replacement of four native promoters in the actinorhodin pathway with four synthetic alternatives with almost 100% efficiency. The multiplex capacity was expanded to allow targeting of six and eight promoter sites with 68 and 32% efficiency, respectively, through the expression of two gRNA sequences from the CRISPR plasmid (Kim et al., 2020). The mpCRISTAR method highlighted that in addition to heterologous gene integrations/deletions or mutations, multiplex approaches could also be used for simultaneous promoter manipulation with high efficiency when CRISPR/Cas9 was employed for DSB induction. Although double-stranded linear DNA fragments were used for promoter replacement in mpCRISTAR, the co-expression of multiple plasmids played an important role in its performance.

### Oligonucleotide-Directed Integration

Oligonucleotide-directed recombination engineering is a method which makes use of small homologous oligonucleotides to hijack native recombination systems. It has been widely applied for multiplex site-directed mutagenesis in bacteria (Warner et al., 2010; van Pijkeren and Britton, 2012). Taking advantage of the highly efficient endogenous homologous recombination machinery in *S. cerevisiae*, an adapted method known as yeast oligo-mediated genome engineering (YOGE) was developed to perform both single and multiplex genome modifications in the species (DiCarlo et al., 2013). However, as single recombination efficiencies were below 1%, repeated iterative transformation cycles were required to enhance recombination and oligo incorporation frequencies (DiCarlo et al., 2013).

**FIGURE 5:**
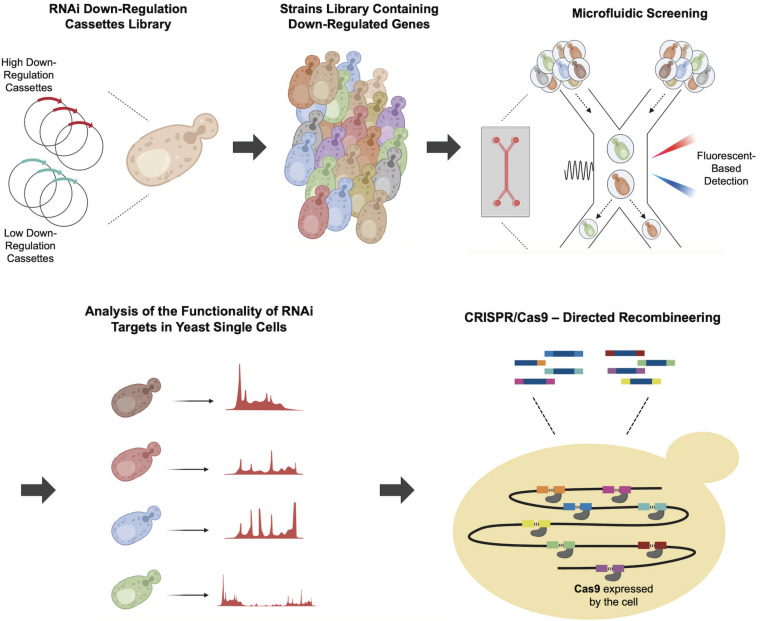
Multiple integration of RNAi cassettes using Cas9-mediated recombineering. To increase recombinant α-amylase production several selected genes, which are responsible for protein synthesis and secretion, were targeted for down-regulation. After introducing the plasmids containing low effect or high effect down-regulation cassettes, the individual cells from the strain library were encapsulated and screened through fluorescent-based microfluidics. Next, the individual cells were analyzed and then, the most efficient combination of genetic perturbations was implemented by CRISPR/Cas9–Directed multiple recombineering into eight regions of the yeast genome, shown with different colors. In each round of transformation, four regions were targeted simultaneously to integrate multiple integration of down-regulation cassettes.

In a more recent application, Wang et al. (2019) developed a CRISPR/Cas9-mediated recombination engineering method to simultaneously integrate multiple RNAi cassettes. The study aimed to improve recombinant protein production by downregulating key genes involved in cellular metabolism, protein modification and degradation, and cell cycle, which are known to have an effect on recombinant protein production. Initially core RNAi machinery genes encoding Argonaute (*AGO1*) and Dicer (*DCR1*) proteins (Drinnenberg et al., 2009; Meng et al., 2017) from *Saccharomyces castelli* were integrated into the genome of a *S. cerevisiae* strain expressing Cas9 to reconstitute the RNAi (Wang et al., 2019). Two sets of RNAi cassettes, featuring high and low down-regulation efficiencies were expressed via plasmids to down regulate several target genes including *YKL222C*, which interacts with ribosomes, *ESBP6*, which encodes a transporter protein and *ULA1*, which plays a role in protein degradation. Microfluidic single-cell screening was subsequently used to select strains harboring the most effective combination of target gene down regulations from the yeast library. The oligonucleotides containing RNAi cassettes were then simultaneously integrated into eight different genomic loci through two rounds of CRISPR/Cas9–mediated recombineering as shown in Figure 5. Following this approach facilitated a 2.2-fold improvement in recombinant α-amylase.

## Pre-Placed Gate Systems

**FIGURE 6:**
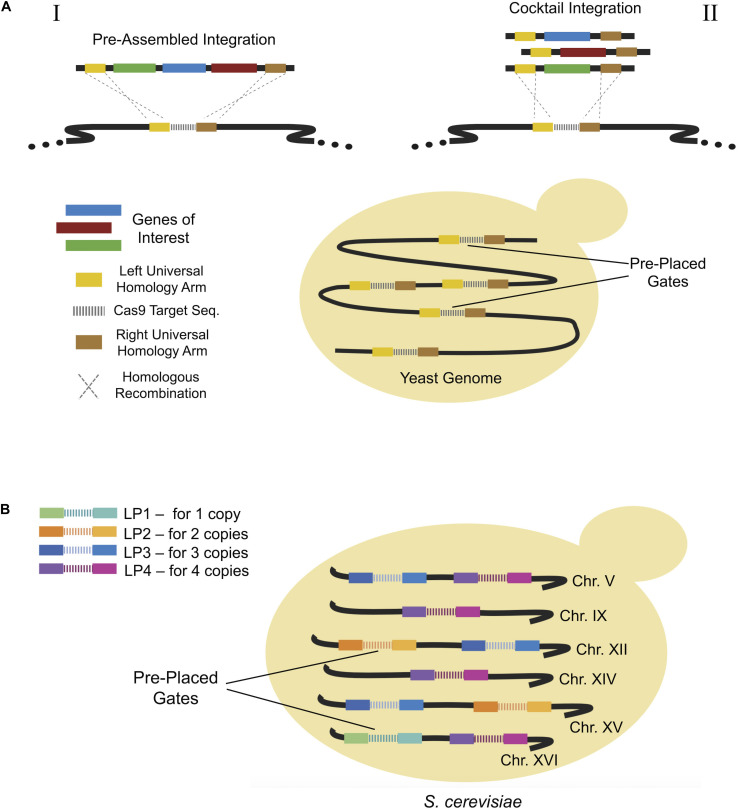
**(A)** Wicket system allowing the integration of multiple pathway genes via universal homologs arms on the target genomic region. Pre-placed synthetic sequences act as gates for the integration of donor DNAs. It is possible to integrate pre-assembled multi genes of a metabolic pathway or to integrate a mix of each single donor DNA simultaneously. **(B)** Landing pad system providing controlled multiple integration of genes of interest. In this approach, a certain number of copies of pre-placed gates is present in the genome so that a particular copy number for each target gene or DNA fragment can be obtained.

Alternative methods for enhancing genome engineering efficiency involve the use of pre-placed synthetic fragments which serve as insertion points or “gates” for exogenous DNA insertion. Hou et al. (2018), constructed strains with multiple copies of small gates known as “wickets” to allow integration of heterologous genes (Hou et al., 2018). Wickets consisted of a gRNA target sequence sandwiched between two universal homology arms (Figure 6A), allowing them to act as multiple integration sites. Using this approach, a β-carotene pathway consisting of three genes, *CrtE*, *CrtI*, and *CrtYB*, was integrated via pre-assembled integration (Figure 6A-I) with almost 100% efficiency in one of four strains used.

Similarly, Bourgeois et al. (2018) constructed a synthetic landing pad system (Figure 6B), which enabled precise multi-copy gene integration between pre-inserted synthetic fragments. Copy number could be carefully controlled using the alternative Landing pad (LP) systems, LP1 for a single copy, LP2 for two copies, LP3 for three copies or LP4 for four copies. After a proof of concept study using GFP, researchers produced (S)-norcoclaurine, a key precursor in benzylisoquinoline alkaloids pathway (Stadler et al., 1989), using the LP system. Different variants of a norcoclaurine synthase (NCS) gene were expressed at various copy numbers, from 1 to 4, using the LP system. This resulted in a maximum (S)-norcoclaurine titer of 130 μg/L, representing a remarkable improvement compared to previous heterologous production attempts (Bourgeois et al., 2018).

The pre-insertion of sequences into the genome allows the multiplex integration of heterologous genes without requiring selective markers thanks to a sandwiched gRNA target region between the sequences. High integration efficiencies can be achieved using such approaches. However, excessive pre-placed integration sites may pose a metabolic burden due to uncontrolled copy numbers. In addition, DSBs on many sequences caused by Cas9 may not always be repaired by the host. Thus, three of four strains used in Wicket system had relatively low integration efficiencies (maximum 50%). Despite this, one colony of the fourth strain harbored over 20 copies of integrated heterologous genes (Hou et al., 2018). The LP system can resolve this bias with controllable copy numbers. Therefore, the exact copy number of target genes can be obtained. The approach could also be adapted to facilitate the integration of more than four copies should higher copy numbers be required.

## Beyond Genomic Integration–Multiplexing for Pathway Optimization

The introduction of heterologous genes into a host organism is key to the construction of heterologous pathways and the production of target products. However, optimization of the resulting constructed pathways and even native pathways is also critical to the development of effective microbial cell factories. Thus, gene deletions, gene disruptions and repression or over-expression of gene expression are important applications within the scope of genome engineering (Ko et al., 2020). As for genomic integration, multiplexing of such applications also has the potential to accelerate the strain development process and CRISPR/Cas9 technologies are also to be proving valuable tools in this context.

**FIGURE 7:**
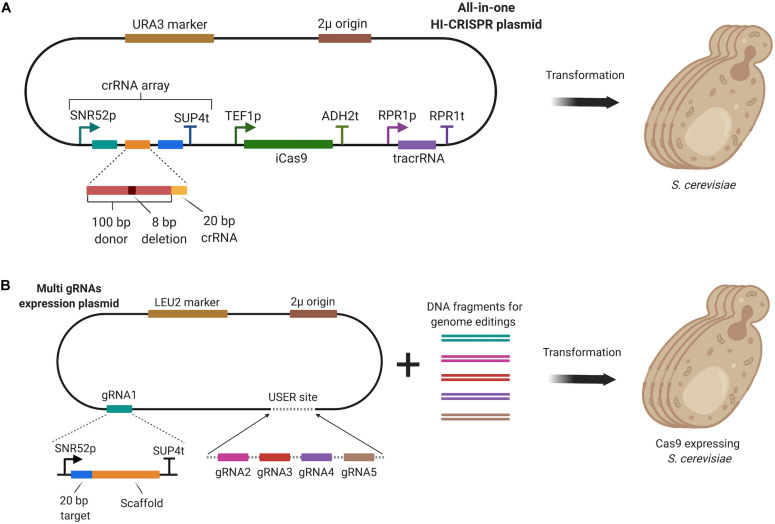
**(A)** HI-CRISPR system, which utilized a single all-in-one vector to express iCas9, the crRNA array, tracrRNA and a mutagenizing donor fragment for multi-gene disruption. **(B)** Multi-gene editing method involving the expression of multi-gRNA cassettes through a plasmid constructed by USER cloning. Cas9 was expressed by the host.

Simultaneous multi-gene disruption in *S. cerevisiae* was first achieved via Homology-Integrated CRISPR-Cas (HI-CRISPR) (Bao et al., 2015). To disrupt the target genes, a homologous 100 bp donor DNA harboring an eight bp deletion and a protospacer adjacent motif (PAM) sequence, was used as mutagenizing fragment, which was inserted between crRNA sequences in the crRNA array. The crRNA array, containing multiple crRNA sequences and disruption donors, and the tracrRNA were expressed under the control of different promoters in a single all-in-one plasmid as illustrated in Figure 7A. After processing of the crRNA array by host nucleases (unknown) and RNase III, tracrRNAs and crRNAs formed a complex to guide an improved Cas9 variant, iCas9, which was discovered during the study. Using the HI-CRISPR approach, triplex disruption of the *CAN1*, *ADE2*, and *LYP1* genes was achieved with 83% efficiency after 4 days of incubation in synthetic dropout medium. In addition, simultaneous disruption of *ATF2*, *GCY1*, and *YPR1* genes was achieved with 100% efficiency after 6 days of incubation.

The HI-CRISPR approach was also employed to disrupt four genes in diploid and triploid yeast strains resulting in eight-allele and twelve-allele deletions, respectively (Lian et al., 2018). Instead of expressing crRNAs like in the HI-CRISPR design, gRNAs were expressed with different copy numbers. With high copy gRNA expression plasmids (∼80 copies/cell), 100% gene deletion efficiency was achieved for four genes, *HIS3*, *TRP1*, *LEU2*, and *URA3* in both a diploid yeast strain (Ethanol Red) and a triploid yeast strain (ATCC 4124).

Although four genes were efficiently disrupted using the HI-CRISPR approach (Lian et al., 2018), gene disruption efficiency decreased dramatically when a target crRNA sequence was placed on the fifth position in crRNA array (Bao et al., 2015). In a later study, however, Jakočinas et al. (2015) were able to overcome this bottleneck when they targeted five different loci in the yeast genome for simultaneous editing to increase mevalonate production. Four genes *BTS1*, *YPL062W*, *YJL064W*, and *ROX1*, were disrupted by incorporating a stop codon on the PAM sequence of the genes. The promoter of a fifth gene, *ERG9P*, was also truncated to downregulate its expression. Individual gRNA expressing cassettes containing their own promoter and terminator sequences were assembled using USER cloning to construct a multi gRNA expression plasmid. Cas9 was then expressed from a separate plasmid (Figure 7B). The researchers reported 100% quintuple genome editing efficiency using the method. Mevalonate production was increased to 10 μM in the resulting optimally engineered strain, representing a 41-fold improvement compared to the wild-type strain (Jakočinas et al., 2015).

**FIGURE 8:**
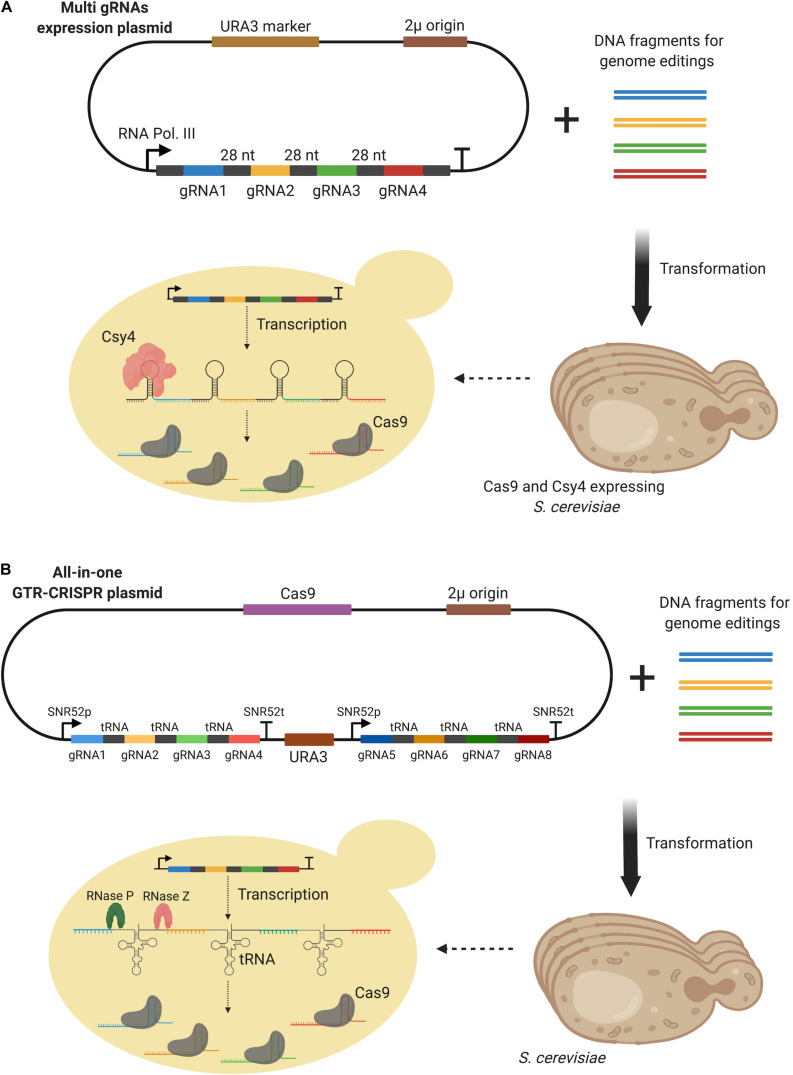
**(A)** Csy4-based multi-gene editing method. gRNAs were expressed from a 2 μ plasmid while Cas9 and Csy4 were expressed by the host. Csy4 cleaved 28 bp stem-loop to release single gRNAs in the host. **(B)** GTR-CRISPR method which benefits from tRNA sequences inserted between gRNAs. Up to eight genes could be edited using this method. All elements, multi-gRNAs and Cas9, were expressed by a single plasmid and endogenous RNase P and RNase Z were used to process tRNA containing transcripts to release gRNAs.

Csy4 is an endoribonuclease of *Pseudomonas aeruginosa* expressed for crRNA biogenesis (Haurwitz et al., 2012). Although it was first employed for the multiplex genome editing of mammalian cells (Nissim et al., 2014), Ferreira et al. (2018) demonstrated its applicability to *S. cerevisiae*. Csy4 was used to increase both the multiple deletions and multiple up-regulation of target genes in *S. cerevisiae*. The endoribonuclease was capable of targeting and cutting a particular stem-loop consisting of a 28 bp nucleotide sequence (Haurwitz et al., 2012). The researchers episomally expressed four gRNAs, with the Csy4 target region sandwiched between them, under the control of a single promoter (RNA Polymerase III promoter, SNR52), in a yeast strain expressing Csy4 and Cas9 as demonstrated in Figure 8A. Quadruple deletion of four genes, *FAA1*, *FAA4*, *TES1*, and *POX1*, was achieved with 96 % efficiency using Csy4, compared to just 50 % efficiency for double gene deletion in the absence of Csy4. A similar approach was also applied for the upregulation of three genome-integrated GFP genes under the control of three different promoters, *HMG1*, *ACS1*, and *OLE1* (Ferreira et al., 2018). However, a dCas9-VPR activator was expressed by the gRNA expressing plasmid, whilst Csy4 was expressed by a second plasmid in the strain. Three different combinations of promoter targeting gRNAs were investigated leading to a two-fold increase in GFP expression compared to those strains not expressing Csy4.

A maximum of eight efficient multi-gene disruptions was recently achieved using a gRNA-tRNA array for CRISPR-Cas9 (GTR-CRISPR) method. Eight genes (*CAN1*, *ADE2*, *LYP1*, *TRP2*, *FAA1*, *FAA4*, *POX1*, *TES1*) were simultaneously disrupted with 87% efficiency when optimal gRNA sequences were used (Zhang et al., 2019). In the GTR-CRISPR design, a tRNA^*Gly*^ sequence was inserted between each gRNA and two SNR52 promoters were used to each transcribe four of the eight gRNAs (Figure 8B). Endogenous tRNAs were processed by two enzymes RNase P and RNase Z, following transcription in a similar way to the Csy4-aided method. A single and complete plasmid including both the multiple gRNAs and Cas9 protein was constructed via Golden Gate assembly. To further streamline the method, an alternative approach, Lightning GTR-CRISPR, was also developed by the researchers. Without pre-assembly of the DNA fragments, the Golden Gate reaction mix was directly transformed into the yeast for *in vivo* assembly. Through the expression of two gRNAs, under SNR52 promoters, 96% and 60% multiple disruption efficiencies were achieved for four (*CAN1*, *ADE2*, *LYP1*, *TRP2*) and six genes (*CAN1*, *ADE2*, *LYP1*, *TRP2*, *FAA1*, *FAA4*), respectively. However, a dramatic decrease in efficiency was reported for the simultaneous disruption of eight genes.

The construction and simultaneous expression of multiple gRNAs are critical to multiplex genome engineering. Jakočinas et al. (2015) constructed a plasmid expressing five different gRNAs using USER cloning. However, as a USER cloning site was first integrated into a plasmid containing a single gRNA expression cassette, an additional step was required for the construction of a multiple gRNA expressing plasmid. This multi-gRNA expressing plasmid was co-transformed with donor DNAs into a Cas9-expressing yeast strain. For the HI-CRISPR method, on the other hand, Bao et al. (2015) made use of Golden Gate dependent assembly to construct an all-in-one HI-CRISPR system which expresses all of the required elements, donor DNA, crRNAs, tracrRNA and Cas9, in a single plasmid. This approach could therefore be applied in a yeast strain without the need for Cas9 expression. In an alternative method, Ferreira et al. (2018) expressed one more element, Csy4, to optimize multiple gRNAs expression and facilitate the expression of all gRNAs using a single promoter. Although the approach demonstrated good potential for the stable expression of multiple gRNAs, a maximum of four gRNAs were simultaneously integrated in the study. Further research is therefore needed to investigate its application for higher numbers of gRNA sequences. The GTR-CRISPR method appears to be the most efficient approach for the simultaneous disruption of large numbers of genes (Zhang et al., 2019). A modified version of this method was also utilized for the deletion of eight genes (four genes per round) in yeast lipid metabolism for increased free fatty acid production. As endogenous tRNA processing enzymes were used and Cas9 was expressed episomally, like HI-CRISPR, this method could theoretically be applied to any yeast strain without the need for genomic integrations. Nevertheless, all of the mentioned approaches present promising solutions for high-efficiency, simultaneous multiple gene disruptions or deletions.

Csy4-aided multiple up-regulation of triple targets was also shown in the aforementioned study (Ferreira et al., 2018). In addition to multiple gene deletions, multiple up-regulation or down-regulation of target genes can be very useful for the fine-tuning of metabolic pathways and production of target products. Lian et al. (2017) developed a tri-functional CRISPR system named CRISPR-AID to simultaneously up-regulate, down-regulate and delete three different target genes in the yeast genome. Researchers integrated homologous donor sequences into gRNA cassettes via the HI-CRISPR method and three CRISPR systems, one for activation, one for interference and one for deletion, were designed. The dCas9-VPR complex was used for up-regulation, dCas9-MXI1 complex for down-regulation and a catalytically active Cas9 was used for deletion. Firstly, the simultaneous five-fold activation of RFP, mCherry, five-fold interference of yellow fluorescent protein, mVenus and deletion of *ADE2* gene with more than 95% efficiency was achieved using the CRISPR-AID method. Production of β-carotene was also increased 2.8-fold by simultaneously overexpressing a *HMG1* gene, downregulating an *ERG9* gene and deleting a *ROX1* gene in a single CRISPR-AID genome engineering step.

Recently, seven genes in *S. cerevisiae* were simultaneously down-regulated (Ni et al., 2019), this is the greatest number of simultaneous down-regulation achieved in a single step in the species. To increase β-amyrin production, *ADH1*, *ADH4*, *ADH5*, *ADH6*, *CIT2*, *MLS1*, and *ERG7* genes were down-regulated using a multi-gRNAs expression plasmid in a yeast strain expressing dCas9 (dead Cas9). A plasmid containing seven cassettes, each expressing one of the gRNAs along with its promoter and terminator, separated by random 20 bp sequences was constructed. Using this method alone, β-amyrin production was increased by 42% to 60 mg/L.

The methods discussed in this section highlight the applicability of multiple genome editing techniques for gene deletion, disruption, up-regulation or down-regulation. Such edits allow significant improvements in the productivity of metabolic pathways to be achieved without further gene integrations. Gene integrations increase both the metabolic burden on the host and the cost of the project. Minimizing the number of gene integrations, whenever possible, through the use of alternative approaches to metabolic pathway optimization is therefore an effective approach.

## CRISPR/Cas12 for Multiplex Genome Engineering

Multiplex genome editing has also been achieved using the alternative, CRISPR/Cas12a method. Verwaal et al. (2018) simultaneously integrated three genes of a β-carotene pathway into the yeast genome using Cas12a (formerly Cpf1). Three crRNAs targeting three different locations within the yeast genome were expressed by a single plasmid in a strain, which episomally expressed Cas12a. Donor DNAs of expression cassettes for the *CrtE*, *CrtYB*, and *CrtI* genes of the β-carotene pathway were included in the transformation mix for crRNA delivery. The three genes were simultaneously integrated with 91% efficiency, facilitating the expression of the pathway. Li et al. (2018) also expressed two different biosynthetic pathways each comprised of three key genes were expressed in *S. cerevisiae* and evaluated their efficiencies. The first of which was a β-carotene pathway comprised of three genes (*CrtE*, *CrtYB*, and *CrtI*) from *Xanthophyllomyces dendrorhous*. A key rate limiting mevalonate pathway enzyme, truncated 3-hydroxy-3-methylglutaryl-coenzyme-A reductase (*tHMG1*) was also expressed. Cas12a and crRNA was expressed from a CEN/ARS plasmid, which was co-transformed with three donor DNA cassettes, *CrtI* (3.6 kb), *CrtYB* (3.8 kb), and *tHMG1-CrtE* (5.5 kb). Triplex integration was successfully achieved with an efficiency of 32%. In order to validate the method, a second pathway was constructed for patchoulol production in *S. cerevisiae*. In the first round of gene manipulation three cassettes were integrated, a farnesyl diphosphate synthase-patchoulol synthase (*FDPS*-*PTS*), *tHMG1* and isopentenyl pyrophosphate isomerase (*IDI1*) with an efficiency of 30%. A second round involving an additional triplex manipulation was subsequently achieved with an efficiency of 30%, enhancing patchoulol titers from 20 to 52 mg/L. A β-Carotene pathway was constructed through multiplex CRISPR/Cas12a in another recent study (Ciurkot et al., 2019). A method was developed for the multiplex integration of *CrtE, CrtYB*, and *CrtI* genes using Cas12a (Ciurkot et al., 2019). In this protocol, a single crRNA expression array comprised of three crRNA units was expressed by a single promoter and terminator in a Cas12a expressing yeast strain. Following transcription of the crRNA array, each of the three crRNA sequences were processed by Cas12a allowing targeting of three different loci within the yeast genome. Triplex integration was achieved with an impressive efficiency of over 90%.

Cas12a from *Francisella novicida* was also used for the simultaneous deletion of four genes, *ADE2*, *CAN1*, *HIS4*, *PDR12*, in *S. cerevisiae* (Swiat et al., 2017). A crRNA array containing a crRNA for each of the four genes mentioned was expressed via a plasmid in two different yeast strains. Of these, one strain, which harbored a chromosomally integrated Cas12a gene, achieved 88% quadruple deletion efficiency. In the other strain, Cas12a was expressed from a multicopy plasmid and simultaneous deletion of the four genes was achieved with 100% efficiency.

Although CRISPR/Cas9 and CRISPR/Cas12 are analogous, there are a number of key differences between the systems. In CRISPR/Cas9 a *trans*-activating crRNA (tracrRNA) forms a duplex with the CRISPR RNA (crRNA). This duplex acts as a guide RNA for the associated Cas9 protein and can be readily programmed to target specific DNA sequences. Cas12 on the other hand is a single RNA guided nuclease and does not require a tracrRNA (Swarts and Jinek, 2018). This reduces the minimum length of RNA sequence required to 42–44 bp, compared to around 100 bp for Cas9 (Zetsche et al., 2015; Adiego-Pérez et al., 2019). Both Cas9 and Cas12 rely on the presence of a PAM. However, where SpCas9 recognizes a 5′-NGG-3′ and less frequently a 5′-NAG-3′ or a 5′-NGA-3′ PAM (Collias et al., 2020), a 5′-TTTV-3′ PAM is typically preferred for Cas12a. Finally, the nature of the DSB induced by the two endonucleases differs with Cas9 producing blunt ends and Cas12 generating overhangs.

Although both Cas9 and Cas12a introduce DSB allowing increased integration efficiency, their PAM sequences and gRNA structures, which are the main factors to be considered for genome engineering, differ. Cas12a has ability to process its own gRNAs as it possesses both endoribonuclease and DNase activity (Swarts et al., 2017; Zetsche et al., 2017). Cas9 on the other hand, relies on additional components to accelerate maturation of multi-gRNAs. For example, RNase P and RNase Z from the tRNA processing system were used to boost the multiplex capacity of Cas9 (Xie et al., 2015). This approach was also used for free fatty acid production in yeast via multiplex genome editing (Zhang et al., 2019). The construction of multi-gRNA assays is also relatively easy using Cas12a as it does not require a tracrRNA. The simpler expression of its crRNAs renders Cas12a a powerful and promising candidate for multiplex genome engineering. On the other hand, the PAM sequence consisting of four bases required by Cas12a may be a limiting factor compared to the shorter three base PAM sequence used by Cas9. Fortunately, it was demonstrated that Cas12a can be engineered to target alternative PAM sequences (Gao et al., 2017) such that its genome targeting range can be increased. These clear advantages indicate that more Cas12a-mediated multiplex genome engineering studies in yeast will emerge in the near future.

## Automated Multiplex Genome Editing

Genome scale multiplex engineering has the potential to generate yeast strains with enormous diversity, however, the availability of effective automation tools for the design, creation and screening of genomic libraries is a major bottleneck (Appleton et al., 2017; Si et al., 2017). In addition, the lack of standardization of methods for microbial genome scale engineering, complicates their development (Beal et al., 2020). Numerous automated workflows have been developed for genome scale engineering in bacteria (Nielsen et al., 2016; Carbonell et al., 2018; El-Mansi et al., 2019; Gao et al., 2019). Wang et al. (2009) developed multiplex automated genome engineering (MAGE), to accelerate the directed evolution of bacteria. In this system, synthetic oligonucleotides were iteratively incorporated into the bacteria where they would bind to the lagging strand of the host during replication and introduce target alteration. The DSB independent MAGE system was capable of performing up to 50 simultaneous genome alterations. However, studies in yeast have been much more limited, largely due to the relative simplicity of *Escherichia coli* and the high efficiency of recombineering based methods in the species at around 30% compared to just 1% in *S. cerevisiae* (Si et al., 2017). Despite this, Barbieri et al. (2017) were able to extend the MAGE method for use in yeast through the development of eukaryotic multiplex automated genome engineering (e-MAGE). Using this, up to 12 oligonucleotide sequences were integrated simultaneously without the need for DSB induction. Although e-MAGE was not a completely automated workflow like the original MAGE, it has the potential for automation.

**FIGURE 9:**
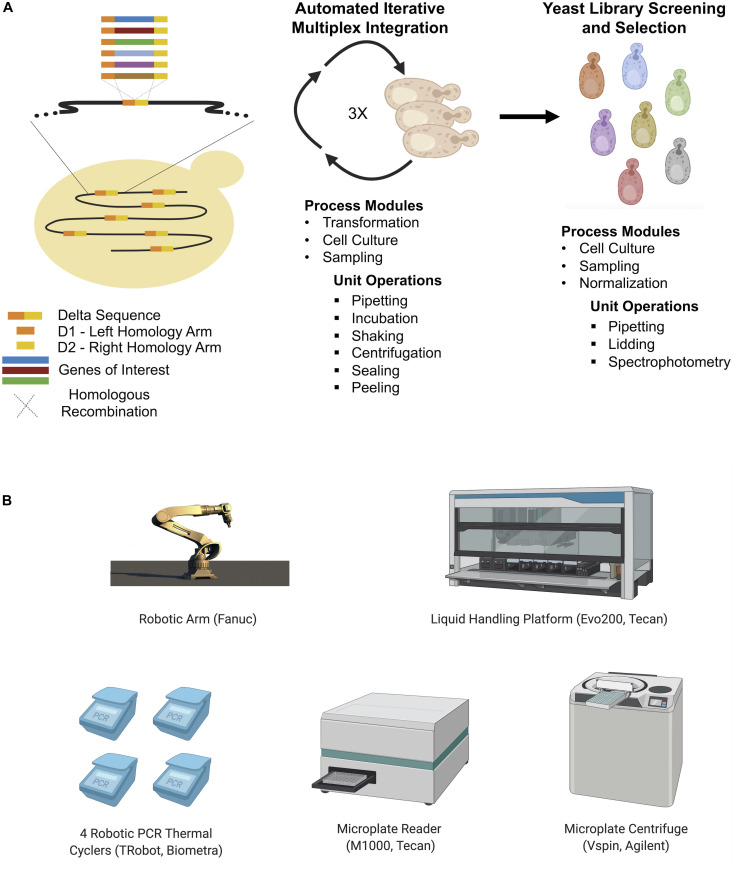
**(A)** General workflow of automated CRISPR-mediated multiplex integration into delta sequences for yeast cell factory development (Si et al., 2017). Yeast library is formed after three rounds of automated transformation and the best strains are selected during a screening and selection step. Process modules represent automated applications in each step (iterative transformation and library screening/selection). Unit operations represent physical operations handled by the automated platform. The transformation process is almost fully automated as most of the transformation steps could be automated while some parts of library screening/selection are automated. Thanks to this automated platform, strains that can express cellulase, produce isobutanol, utilize glycerol, and have acetic acid tolerance were developed faster than manual equivalents. **(B)** The main hardware used in the described automation process. Additionally, an automated de-lidding station, a plate sealer and seal peeler were also used to support the automation process.

In the absence of selection markers, recombineering is limited to short oligonucleotides modifications, which may be insufficient to modulate gene expression in more complex yeast species (Si et al., 2017). Si et al. (2017) developed an alternative automated multiplex yeast genome engineering method. Initially an optimized CRISPR/Cas9-assisted multiplex delta integration workflow was constructed using green fluorescent protein (GFP). An automated multiplex genome-scale engineering system comprised of a central robotic platform and a modular computational framework was subsequently employed to enhance the acetic acid tolerance (HAc) of *S. cerevisiae*. The resulting HAc resistant strains were capable of growing stably in the presence of 1.1% HAc, a condition which completely inhibited growth of the parent strain. This was achieved via automated iterative integration using standardized CRISPR/Cas9 delta integration (Figure 9). To build the automated platform, a workflow defining the whole process from the primary cultivation of the target strain to glycerol stock preparation for the optimized engineered strain was translated into an executable sequence of unit operations. Such operations included thermocycling, liquid handling, centrifugation, incubation, and spectrophotometric measurements. The resulting programmed operation was implemented using the computational framework and robotic system. Thus, an entirely automated system with a standardized workflow involving cloning, protein engineering, pathway construction, genome engineering, strain library screening, evolutionary engineering, and genotyping was successfully used to engineer a strain with desirable traits.

Apart from automation hardware and software to accelerate strain development, cutting-edge computational modeling approaches and design tools are being increasingly implemented to aid the design of pathway variants. For example, web-based gRNA design tools such as CRISPOR or CHOPCHOP (Concordet and Haeussler, 2018; Labun et al., 2019) can now be used to select appropriate gRNA candidates on a specific region. The software mentioned not only provide an easy to use GUI but also present efficiency scores and the probability of off-target effects of the gRNA sequences, significant factors for multiple genome engineering, predicted by several computational methods (Moreno-Mateos et al., 2015; Doench et al., 2016). Such technologies have alleviated major bottlenecks in the design and build phases of cell factory development.

## Multiple Genome Editing Studies About Non-Conventional Yeast Species

Although most multiplex yeast genome editing studies typically have involved the model *S. cerevisiae* species, the methods are increasingly being extended to applications involving non-conventional yeasts (Delic et al., 2013; Horwitz et al., 2015; Holkenbrink et al., 2018; Shi et al., 2018). *Y. lipolytica* is an emerging industrially important non-conventional yeast platform for the production of fine chemicals (Shi et al., 2018). The CRISPR mediated EasyClone method was recently adapted for use in *Y. lipolytica* (Holkenbrink et al., 2018). The method termed, EasyCloneYALI, was used to integrate five vectors into the genome of the strain. Multiple gRNA sequences targeting various regions in the *Y. lipolytica* genome were employed. Successful integration of the five vectors was confirmed via colony PCR in over 80% of transformants. A triplex gene disruption involving *TRP1*, *PEX10*, and *GUT2* genes was also studied in *Y. lipolytica* (Gao et al., 2016). Although the average efficiency of the triplex gene disruption was relatively low at 19%, the simultaneous duplex disruption efficiency of *TRP1* and *PEX10* was higher at 37%.

The methylotrophic yeast, *Pichia pastoris* (*Komagataella* spp.) is widely used for recombinant protein production and is a promising chassis for production of valuable biochemicals (Gasser and Mattanovich, 2018). CRISPR-mediated multiple loci integration was recently performed in *P. pastoris* (Liu Q. et al., 2019). Through the expression of multiple gRNA sequences, duplex and triplex integration of an eGFP coding gene was achieved in the strain with 70 and 32% efficiency, respectively. A biosynthetic pathway for 3-methyl catechol production comprised of three genes was also constructed in *P. pastoris* through a single-step integration (Gasser and Mattanovich, 2018). In addition, multiplex gene deletion has been performed in *P. pastoris*, the simultaneous deletion of genes *GUT1* and *AOX1* was achieved using CRISPR/Cas9 with 69% efficiency (Weninger et al., 2016).

*Kluyveromyces lactis* (*Kluyveromyces marxianus*) is another important yeast species used in food and feed industries due to its ability to metabolize lactose and effective protein secretion mechanism (Cai et al., 2019). The modular gRNA delivery approach developed in *S. cerevisiae* was also applied in *K. lactis* (Horwitz et al., 2015). As in *S. cerevisiae*, a muconic acid pathway consisting of six genes totaling 9.7 kb distributed across three cassettes were integrated into the genome of *K. lactis* (Horwitz et al., 2015). Although muconic acid production was successfully achieved in *K. lactis*, the multiplex integration efficiency was relatively low at around 2%. In another study a double gene inactivation was performed in *K. lactis* (Cernak et al., 2018). The genes *ALPHA3*, and *KAT1*, which are responsible for mating-type switching were simultaneously inactivated by using CRISPR/Cas9 generating stable heterothallic haploids (Cernak et al., 2018).

## Discussion

A wide range of techniques have been developed for *S. cerevisiae* multiplex genome engineering and microbial cell factory development. The flexibility and specificity of CRISPR/Cas has proven effective in enhancing the efficiency of wide-ranging multiplex genome editing techniques. This review revealed factors such as copy number, donor DNA number, and integration efficiency are of great importance in multiplex genome editing. Table 1 summarizes the discussed yeast multiple integration methods in terms of such parameters.

**TABLE 1:** General review of different multiplex integration methods in terms of donor number, copy number, integration size, and integration efficiency.

**Method Used**	**Number of Integrated Donor DNAs**	**Copy Number of Integrated DNAs**	**Total Size of Integration**	**Integration Efficiency**	**Yeast Species**	**References**
CRISPRm	3	1	200 bp<	<20%	*S. cerevisiae*	Ryan et al., 2014
Modular gRNA Delivery	3	1	>200 bp	64%	*S. cerevisiae*	Horwitz et al., 2015
Modular gRNA Delivery	6	ranges from 1 to 5	24 kb	<5%	*S. cerevisiae*	Horwitz et al., 2015
Conventional delta (d) integration	2	ranges from 3 to 5	ranges from 7 to 12 kb	N/A	*S. cerevisiae*	Sakai et al., 1990
Di–CRISPR	7	≤18	≤432 kb	>70%	*S. cerevisiae*	Shi et al., 2016
CRISPR-mediated delta integration	4	≤25	>100 kb	ranges from 50 to 70%	*S. cerevisiae*	Huang and Geng, 2020
CRITGI	3	N/A	>15 kb	10%	*S. cerevisiae*	Hanasaki and Masumoto, 2019
CRITGI	1	≤12	>5 kb	75%	*S. cerevisiae*	Hanasaki and Masumoto, 2019
Conventional integration into rDNA cluster	1	≥100	ranges from 1000 to 2000 kb	N/A	*S. cerevisiae*	Lopes et al., 1989
CMGE-MC	1	≤10	≤15 kb	46%	*S. cerevisiae*	Wang et al., 2018
Combination of δ-integration and rDNA integration	3 (1+2)**	4	∼24 kb	N/A	*S. cerevisiae*	Park and Hahn, 2019
CRISPR-mediated integration into rDNA cluster	1	∼150	∼8.5 kb	N/A	*S. cerevisiae*	Chiou and Armaleo, 2018
Plasmid-based multiple integration	3	1	∼3.8 kb	44%	*S. cerevisiae*	Jensen et al., 2014
CrEdit	3	1	17.5 kb	84%	*S. cerevisiae*	Ronda et al., 2015
EasyCloneYALI	5	1	>6 kb	80%	*Y. lipolytica*	Holkenbrink et al., 2018
mpCRISTAR	6	1	∼3 kb	68%	*S. cerevisiae*	Kim et al., 2020
mpCRISTAR	8	1	∼4 kb	32%	*S. cerevisiae*	Kim et al., 2020
Wicket	3	ranges from 2 to 5	ranges from 12 to 14 kb	100%	*S. cerevisiae*	Hou et al., 2018
Landing Pad	1	3	∼4.5 kb	53%	*S. cerevisiae*	Bourgeois et al., 2018
Landing Pad	1	4	∼6 kb	39%	*S. cerevisiae*	Bourgeois et al., 2018
Multi-loci Integration in *Pichia pastoris*	3	1	∼4.5 kb	32%	*P. pastoris*	Liu Q. et al., 2019
Modular gRNA Delivery	3	1	9.7 kb	2%	*K. lactis*	Horwitz et al., 2015
CRISPR/Cas12a	3	1	∼9 kb	∼91%	*S. cerevisiae*	Verwaal et al., 2018
CRISPR/Cas12a	3	1	∼12 kb	32%	*S. cerevisiae*	Li et al., 2018
CRISPR/Cas12a	3	1	ranges from 3 to 4 kb	ranges from 50 to 94%	*S. cerevisiae*	Ciurkot et al., 2019

**First donor DNA was integrated by delta integration yielded four copies, then two donor DNAs were integrated into the rDNA cluster yielded four copies for each donor.

For yeast microbial cell development numerous multiplex genome editing tools are available and the most appropriate method is highly application dependent. According to Table 1, the rDNA cluster method is superior for high-copy number integrations. However, in the four studies covered vast deviations were observed in the copy numbers achieved through targeting rDNA clusters. In the original rDNA clusters aided multiplex integration study, successful integration of between 100 and 200 copies of heterologous genes was confirmed by restriction analysis (Lopes et al., 1989). However, a recent study employing this method reported a heterologous gene copy number of just four (Park and Hahn, 2019). Another study coupling the rDNA cluster method with the highly efficient CRISPR/Cas9 system reported a maximum copy number of ten (Wang et al., 2018). In both cases, copy number was validated via qPCR, a much more advanced and precise method compared to restriction analysis. Chiou and Armaleo (2018), claimed to simultaneously integrate introns into all 150 rDNA clusters within the *S. cerevisiae* genome. Intron integration was validated using a PCR-based technique on three strains as qPCR cannot be used for intron copy number detection. A negative control targeting an intronless region was included and no negative bands were detected suggesting all rDNA copies had been successfully modified. It is possible that differences in validation method could be responsible for inconsistencies between copy numbers as well as additional parameters such as the size and delivery efficiency of donor DNAs and Cas9 activity. Another method suited to high-copy number integration is CRISPR mediated δ-integration. This method was used in two recent studies to integrate multiple genes in to δ sites, yielding 18 (Shi et al., 2016) and 25 copies (Huang and Geng, 2020), respectively.

High heterologous gene copy number is not always desirable in the development of effective cell factories, however, as it can increase metabolic burden and decrease cell fitness. Park and Hahn (2019) performed multi-copy integration of the key *ILV3* and *ILV5* genes into rDNA clusters to produce isobutanol. Cultivation of a strain possessing three copies of the genes produced a higher isobutanol titer than a strain with four copies (Park and Hahn, 2019). Pre-placed gate systems offer a solution for controlling copy number, the Landing Pad method for example, allows integration with a precise number of copies ranging from one to four (Bourgeois et al., 2018). Such systems may be preferable for the fine-tuning of metabolic pathways. Pre-placed sequences also offer high integration efficiency as the simultaneous integration of three genes was achieved with almost 100% efficiency (Hou et al., 2018). This was attributed to the method facilitating the use of designed synthetic sequences as target regions rather than native genomic regions. Such systems allowing precise control of copy numbers, reliable high integration efficiency and gene expression can greatly contribute to the standardization of synthetic biology and more specifically of multiplex genome engineering tools.

## Conclusion

In this review recent innovative studies regarding multiplex yeast genome engineering methods were compiled. Numerous approaches were considered with the most appropriate depending on the specific end goal. The repertoire of techniques and approaches is rapidly diversifying and strengthening not only for Baker’s yeast but also for other industrially important yeast species. Improvements in omics technologies and the implementation of computational approaches to biology further boost the development of effective multiplex genome engineering techniques.

Traditional yeast specific multiplex integration techniques include δ-integration and integration into rDNA clusters. Although effective, such methods are often hindered by relatively low integration efficiencies which cannot support an effective multiple integration. Coupling these yeast specific approaches with the highly flexible and efficient CRISPR/Cas system was shown to greatly enhance integration efficiency up to almost 100% across a range of applications. Pre-placed gate systems are other useful alternatives that can be coupled with CRISPR/Cas technology to achieve multi-copy integrations into pre-determined regions in the genome. Apart from multiple integrations, multiple gene disruption/deletion, up-regulation and down-regulation can also be multiplexed through the expression of multiple gRNAs and coupling with Cas9 or dCas9. Although Cas9 is the most ubiquitously used endonuclease in CRISPR systems, alternative endonucleases such as Cas12a have also proven beneficial in multiplex genome editing studies. With different PAM sequences, such various endonuclease options allow greater regions to of the yeast genome to be selectively targeted. In addition, shorter gRNA sequences are required for Cas12a, making it a promising candidate for multiplex studies.

High-throughput automation tools play an important role in expediting the Design Build Test Learn cycle and improving reproducibility in synthetic biology (Jessop-Fabre and Sonnenschein, 2019). Substantial improvements in omics technology, high-throughput cloning and DNA assembly tools and computational capacity has rendered the implementation of these innovative multiplex engineering methods relatively straightforward. Through coupling automated liquid handling tools with the CRISPR-mediated multiplex techniques discussed in this study, large combinatorial strain libraries can be constructed in a high-throughput manner.

## Author Contributions

KM and LR-S conceived the work. KM and LW wrote the manuscript. KM prepared the figures. LR-S assisted with writing, editing, and finalizing the manuscript. All the authors have read and approved the final version of the manuscript.

## Conflict of Interest

The authors declare that the research was conducted in the absence of any commercial or financial relationships that could be construed as a potential conflict of interest.
